# Information, Education, and Communication Services in MCH Care Provided at an Urban Health Center

**DOI:** 10.4103/0970-0218.58386

**Published:** 2009-10

**Authors:** Bratati Banerjee

**Affiliations:** Department of Community Medicine, Maulana Azad Medical College, New Delhi - 110 002, India

**Keywords:** Child care, IEC, MCH care

## Abstract

**Background:**

Regular IEC programs during antenatal and intranatal period, through individual or group approach, brings desirable changes in health practices of people, resulting in a healthy mother and a healthy baby.

**Materials and Methods:**

This study was conducted to assess the level of IEC services regarding pregnancy and child care, received by the women at an MCH clinic of an urban health center, where the study subjects comprised 400 antenatal (AN) and postnatal (PN) women and mothers of children under five years.

**Results:**

Warning signs of danger was explained to only 10% of the AN and PN women. Advice regarding family planning appeared to be the most frequently covered, though that too was explained to less than half of the subjects. About one third of the women were advised on breast feeding. Only 8% of the mothers had been told about all issues regarding pregnancy and child care. Breast feeding and weaning was properly explained to 85.7 and 81.1% of the total mothers of U5 children. Advice regarding subsequent nutrition was given to 60.9% of mothers. About only a quarter of the total mothers were advised on home management of diarrhea and acute respiratory infections. Very few mothers were counseled about the growth pattern of the children and none were shown the growth chart. Only 12.9% of the mothers were informed about all issues.

**Conclusion:**

IEC regarding maternal and child care other than feeding practices is a neglected service in the health facility where the study was conducted.

## Introduction

Health of a child is determined from the prenatal period through infancy to childhood. Poor health of the mother and complications during pregnancy has profound impact on the health of the child. Care should therefore start from the antenatal period of the mother and child care should continue till they become five years. In addition to clinic-based care, mothers should be enlightened on home care and healthy practices, and also recognition of early danger signs.

In reproductive and child health (RCH) program and subsequently the integrated management of neonatal and childhood illnesses (IMNCI), information, education and communication (IEC) have specific role to play for bringing desirable changes in health practices of people. This requires the maternal and child health (MCH) functionaries to regularly undertake IEC programs, through individual or group approach. In this context, a study was designed to assess the level of IEC services regarding pregnancy and child care, received by the women at an MCH clinic of an urban health center (UHC).

## Materials and Methods

A community-based, cross-sectional, observational study was undertaken over a period of six months, in an UHC of Kolkata that caters to a mixed population of 1.18 lakhs from all sections of the community. The study subjects comprised 400 antenatal (AN) and postnatal (PN) mothers and mothers of children under five (U5) years.

As the IEC services involved multiple issues, no consolidated data could be found from past literature about proportion of women receiving such services. Therefore, to determine sample size, *P* was taken as 0.5, considering the theory of probability that 50% are likely to have received the services, which also gives the maximum sample size. Thus, sample comprised 400 women, considering 95% confidence interval and allowing 10% error.

The number of U5 children was noted from the records, and the estimated number of AN and PN mothers at one point of time were calculated from the birth rate and population of the area during the previous year.(1) To determine the size of these different groups within the sample, approximately 15% of the total population in each group was taken. Thus, the sample comprised 350 U5 children and 50 AN and PN mothers. Only one person was selected from each household if more than one eligible participant was found.

For selecting the sample, the households were used as the sampling units. Multistage sampling was done, one of the four units of the UHC serving the population being selected by simple random technique using lottery method in the first stage, and the requisite number of households, that is, 400, of the selected units being identified by systematic random sampling method from the family folders in the second stage. In case of nonavailability of eligible participant in the selected household, the next was considered till the desired sample size was obtained. Data was collected by house visits and interview method, using a pre tested, semi structured schedule which included IEC services, received by the mothers, regarding important points about pregnancy and child care, in the AN and PN period. Proportion of women receiving such services regarding issues under consideration was analyzed.

## Results and Discussion

The word ‘behavior’ refers to peoples' and communities' existing knowledge, opinions, attitudes, and practices for their health and its care. Peoples' health behavior changes over time through the process of acquiring new information and knowledge (awareness) about their health and its care, which lead them to form their opinion, attitudes (favorable and unfavorable) and acceptance and rejection in real life situations. The changes in peoples' behavior also require transmitting and sharing RCH information, which improves the clients' level of knowledge and scientific attitude toward health care and health services. This also motivates other people in the community to adopt new RCH care practices with resultant improvement in the health status of mothers and children.(2) Unfortunately, this still remains as a neglected component in all programs.

In the present study, less than half percentage of AN and PN women received IEC regarding any issue. Advice regarding family planning appeared to be the most frequently covered, though that too was only 42%, that is, less than half of the subjects. About one third of the women (34%) were advised on breast feeding their children. Warning signs of danger in pregnancy was explained to 10% of the AN and PN women. Only 8% of the mothers had been told about all issues regarding pregnancy and child care [Table 1]. Pandit *et al*, in their study center, observed that the IEC activities were of rudimentary nature.(3) In a study carried out in Lucknow, IEC about danger signs was given to only 2.2% and about breast feeding to 70%. To 80% of the clients contraceptive options were described, but their side effects to only 7.7%. Almost 50-60% of clients were told about the correct usage and the source of supply.(4) A comparative study carried out in several cities revealed that knowledge of all family planning methods and awareness of spacing methods was considerably higher among the slum dwellers of Kolkata, than in other places.(5)

**Table 1:** IEC received by the AN and PN women during their antenatal period

Issues	Number of women (n =50)	Percentage (%)
Minor ailments	9	18.0
Warning signs	5	10.0
Breast feeding	17	34.0
Family planning	21	42.0
All issues	4	8.0

IEC services regarding all issues of child care other than feeding practices was covered in only a quarter or less percentage of subjects. Breast feeding and weaning was properly explained to 85.7 and 81.1% of the total mothers of U5 children respectively. Advice regarding subsequent nutrition was given to 60.9% of mothers. About a quarter of the total mothers were advised on home management of diarrhea and acute respiratory Infections (ARI). Very few mothers were counseled about growth pattern of the children and none were shown the growth chart. Only 12.9% of the mothers were informed about all issues [Table 2]. In a study on immunization awareness, on an average less than a quarter of mothers were found to have the desirable level of awareness of 80% or more.(6) Lack of information was the main reason for nonimmunization in almost two thirds of the children, in another study performed in Delhi slums,(7) Bajaj observed that awareness regarding child care in Delhi slums ranged from 52 to 82%.(8)

**Table 2:** IEC received by the mothers of U5 children regarding child care

Issues	Number of women (n =350)	Percentage (%)
Growth pattern	70	20.0
Breast feeding	300	85.7
Weaning	284	81.1
Nutrition	213	60.9
Diarrhea	97	27.7
ARI	84	24.0
ICDS services	61	17.4
All issues	45	12.9

Low awareness among the clients is one of the major reasons of low utilization of services. The MCH strategy of government focuses on the increase of knowledge and utilization of MCH services by all segments of the population. A study conducted in five Delhi slums revealed that the women who had not availed of care during pregnancy said that they either “did not know” about the services or “did not feel the need” to use them.(8) This reflects the need for disseminating proper message, so as to improve the knowledge and utilization of MCH services, which will ultimately have its impact on the health status of the vulnerable section of the community.

## Conclusion

IEC regarding maternal and child care other than feeding practices is thus seen to be a neglected service in the health facility where the study was conducted.
